# Axonal damage in the making: Neurofilament phosphorylation, proton mobility and magnetisation transfer in multiple sclerosis normal appearing white matter^☆^

**DOI:** 10.1016/j.expneurol.2011.09.011

**Published:** 2011-12

**Authors:** A. Petzold, D.J. Tozer, K. Schmierer

**Affiliations:** a UCL Institute of Neurology, Dept. of Neuroinflammation, Queen Square, London WC1N 3BG, UK; b MS Centre Amsterdam, VU University Medical Centrum, Amsterdam, The Netherlands; c Blizard Institute, Centre for Neuroscience and Trauma (Neuroimmunology Group), Barts and The London Queen Mary School of Medicine and Dentistry, London E1 1BB, UK

**Keywords:** AL, acute lesion, BSP, brain-specific proteins, CDP, chronic disease progression, CNS, central nervous system, CTRL, control group, EDSS, Expanded Disability Status Scale, ELISA, enzyme linked immunoabsorbent assay, GM, grey matter, IQR, interquartile range, MS, multiple sclerosis, MRI, magnetic resonance imaging, MTR, magnetisation transfer ratio, NLWM, normal lesional white matter, Nf, neurofilament, NfH, neurofilament heavy chain, PP, primary progressive, RR, relapsing remitting, SAPK, stress-activated protein kinase, SP, secondary progressive, WM, white matter, Neurofilament phosphoforms, Biomarker, MRI, MTR, Multiple sclerosis, Axonal injury

## Abstract

**Aims:**

Multiple sclerosis (MS) leaves a signature on the phosphorylation and thus proton binding capacity of axonal neurofilament (Nf) proteins. The proton binding capacity in a tissue is the major determinant for exchange between bound and free protons and thus the magnetisation transfer ratio (MTR). This study investigated whether the MTR of non-lesional white matter (NLWM) was related to the brain tissue concentration of neurofilament phosphoforms.

**Methods:**

Unfixed post-mortem brain slices of 12 MS patients were analysed using MTR, T1 at 1.5 T. Blocks containing NLWM were processed for embedding in paraffin and inspected microscopically. Adjacent tissue was microdissected, homogenised and specific protein levels were quantified by ELISA for the Nf heavy chain (NfH) phosphoforms, glial fibrillary acidic protein (GFAP), S100B and ferritin.

**Results:**

Averaged hyperphosphorylated NfH (SMI34) but not phosphorylated NfH (SMI35) levels were different between individual patients NLWM. The concentration of hyperphosphorylated NfH-SMI34 correlated with T1 (R = 0.70, p = 0.0114) and — inversely — with MTR (R =−0.73, p = 0.0065). NfH-SMI35 was not correlated to any of the MR indices.

**Conclusions:**

Post-translational modifications of axonal proteins such as phosphorylation of neurofilaments occur in NLWM and may precede demyelination. The resulting change of proton mobility influences MTR and T1. This permits the in vivo detection of these subtle tissue changes on a proteomic level in patients with MS.

## Highlights

► Neurofilaments are phosphorylated ► Protein phosphorylation competes with the free proton binding capacity ► Magnetisation transfer depends on magnetisation exchange between macromolecular bound and free protons ► In multiple sclerosis the phosphorylation status of neurofilaments is changed in otherwise normal appearing axons ► In vivo assessment of early axonal pathology is possible using magnetisation transfer

## Introduction

Multiple sclerosis (MS) is an inflammatory, demyelinating disease of the central nervous system (CNS) with associated neurodegeneration (Lassmann et al., 2007; Trapp and Nave, 2008). The most obvious pathological finding in MS brain tissue is focal demyelination, which may affect any part of the CNS (Chard and Miller, 2009). New MS lesions may result in neurological symptoms and/or signs, a clinical situation called “relapse”, though patients with non-relapsing (primary or secondary progressive) MS also develop new lesions, albeit less frequently (Petzold, 2008).

It is well known that areas of focal demyelination (MS lesions) represent only a fraction of MS pathology, with additional contribution of non-lesional changes of brain parenchyma (Bjartmar et al., 2001; Howell et al., 2010; Kutzelnigg et al., 2005; Zeis et al., 2008) and meninges (Magliozzi et al., 2007). Both lesional and non-lesional MS brain parenchyma is affected by axonal damage (Albert et al., 2007; Kutzelnigg et al., 2005; Peterson et al., 2001; Petzold, 2005; Trapp and Nave, 2008). Neurodegeneration is the major cause of irreversible neurological disability in patients with MS (Trapp and Nave, 2008). The evolution of axonal loss, however remains enigmatic (Moore et al., 2000). This is partly because axonal damage in people with MS is difficult to visualise *in vivo* at a resolution conducive to the investigation of underlying mechanisms.

Magnetisation transfer (MT) is a quantitative magnetic resonance imaging (MRI) technique, based on the exchange of magnetisation between protons in at least two pools: mobile protons and protons bound to macromolecules (Tofts et al., 2003). Changes in MT derived measures including the MT ratio (MTR) in the CNS of patients with MS have been considered to reflect changes in the amount of myelin (Barkhof et al., 2003; Schmierer et al., 2007). This has clearly been shown in studies combining lesional and non-lesional brain tissue (NLBT). The extent to which tissue changes may be detectable with MTR applied to NLBT alone is less well known. In this study we investigated the association between MTR and proteomic alterations of axons in MS non-lesional white matter (NLWM).

Based on histological studies it is well accepted that damage already occurs to axons in the NLWM (Lovas et al., 2000; Trapp et al., 1998). The typical picture is that of irregular changes of the axonal diameter and axonal end bulbs (Trapp et al., 1998). Immunohistochemical studies suggest that protein phosphorylation increases, at least for the neurofilament heavy chain (NfH) and tau (Anderson et al., 2008; Petzold et al., 2008; Schneider et al., 2004). These histological and immunohistochemical data are consistent with changes on a proteomic level (Dutta et al., 2006; Herrero-Herranz et al., 2008; Petzold et al., 2008). Protein phosphorylation appears to be an important biochemical mechanism by which axonal injury is mediated (Petzold et al., 2008). Many of the well established mechanisms driving neurodegeneration (glutamate excitotoxicity, mitochondrial failure) are related to Ca^2 +^ influx, activation of kinases and phosphorylation of NfH (Akassoglou et al., 2002; Brownlees et al., 2000; Grant and Pant, 2000; Petzold, 2005; Schwarzschild et al., 1999; Xia et al., 1996).

An increase of NfH phosphorylation changes the charge depended proton binding capacity of the protein. Non-phosphorylated NfH only binds two protons (Chang et al., 2009). Phosphorylated NfH in contrast has the ability to bind up to 82 protons, a remarkable difference (Chang et al., 2009). The biological relevance of NfH phosphorylation in the MS brain is not known. On a proteomic level phosphorylation of NfH causes radial extension of sidearms out of the dense polymer structure. This is explained by charge repulsion and locally altered entropic and electrostatic interactions of the neurofilament network (Kim et al., 2011; Petzold, 2005). Here we report preliminary evidence that changes in the phosphorylation status of NfH, and hence the proton binding capacity of the neurofilament network, is detectable using MT imaging in MS NLWM.

### Material and methods

This study was approved by the Joint Ethics Committee of the UCL Institute of Neurology and The National Hospital for Neurology & Neurosurgery, London, UK. The MRI data sets and paraffin embedded tissue blocks were used alongside snap frozen samples of NLWM obtained from the brain tissue in unfixed condition. The brain slices had been donated by 10 women and 2 men with MS to the UK Multiple Sclerosis Tissue Bank (MSTB) based at Imperial College London. Brain slices used were collected from the MSTB within a mean of 17 (SD = 6) hours post mortem. Demographic data including age, disease duration, course and brain weight were obtained from the case records collected at the MSTB and based on information from the same source. Disability was estimated using the expanded disability status score (EDSS) scale (Kurtzke, 1983). Some of the MRI data sets used in this study had been used previously to investigate changes of MRI data in the white matter following fixation (Moore et al., 2000; Schmierer et al., 2008).

### Tissue handling

In each case a coronal brain slice (≈ 1 cm thick) from one hemisphere had been dissected at the level of the mamillary bodies, sealed in a plastic bag and stored in a refrigerator at 2–8 _?_C. Three hours before MRI brain slices were taken out of the fridge, carefully wrapped in polyethylene film and left to warm up to scanner room temperature (≈ 20? C). Immediately after MRI, samples of NLWM (size = 0.5–1 cm^3^) were obtained from the brain slices and snap frozen in liquid nitrogen. Digital images of the brain slices were taken showing the location where snap frozen samples had been obtained. Thereafter, brain slices were immersed in 10% buffered formalin.

### MRI scanning and parameter map calculation

Scans were acquired on a GE Signa Horizon Echospeed 1.5 T system (General Electric, Milwaukee, WI, USA) using a birdcage head coil. The MRI plane was positioned parallel to the coronal surface and in the centre of each brain slice. The following data sets were acquired using an imaging slice thickness of 5 mm, a field of view (FOV) of 24 × 24 cm^2^ and a matrix size of 256 × 256 (giving a pixel size of 0.94 × 0.94 mm^2^):

• 2D dual spin-echo (SE) proton density (PD)- and T_2_-weighted (T_2_w) images with parameters TR = 2000 ms and TE = 30/120 ms, respectively.
• 2D PD and T_1_w gradient echo images TR/TE/flip angle = 1500 ms/11 ms/45? and 36 ms/11 ms/45 _?_, respectively), from which T_1_ maps were generated as previously described (Moore, 2003).
• 2D dual SE images (TR/TE1/TE2 = 1720 ms/30 ms/80 ms) obtained with (M_*sat*_) and without (M_0_) a sinc shaped saturation prepulse applied 1 kHz off water resonance, from which MTR maps were calculated according to MTR = 100 × (M_0_−M_*sat*_)/M_0_ (Schmierer et al., 2007).

### Definition of ROI

All scans and maps were displayed on a Sun workstation (Sun Microsystems, Mountain View, CA, USA) using DispImage (Grimaud et al., 1996; Plummer, 1992). Areas of NLWM were defined on T2w SE images as areas of white matter that was free from hyper-intense signal suggesting MS lesions. The absence of lesions in areas where snap frozen samples had been obtained was confirmed by visual inspection of the T2w scans using the digital images of brain slices obtained in the dissection theatre as a reference. Accuracy of the correspondence between areas of NLWM detected on MRI and their histological substrates in brain slices were further enhanced through the use of a previously described stereotactic procedure in all cases (Schmierer et al., 2003).

### Pathological procedures

Tissue blocks sized approximately 1.5 × 1.5 × 1 cm and containing the areas of NLWM detected on MRI were dissected. The blocks were cut in half using a 5 mm deep iron angle resulting in two blocks of approximately 5 mm thickness each with the cutting plane corresponding to the centre of the MRI plane. Blocks were processed for embedding in paraffin and sections stained for haematoxylin & eosin (H&E), Luxol-Fast blue (LFB) and Bielschowsky's silver impregnation. Sections were inspected to confirm no areas of demyelination or remyelination (Brück et al., 2003; Schmierer et al., 2004) had been sampled for protein extraction (see below).

### Protein extraction

Snap-frozen blocks of brain tissue from MS cases were cut and re-suspended at 1:5 g/mL in Tris-HCl buffer (100 mM Tris, pH 8.1 with 1% Triton X-100). A protease inhibitor cocktail (Sigma, P 8340) was added in a dilution 1:100. Samples were homogenised on ice by sonication, triturated 3 times through 19 and 21 gauge needles and spun at 20,000 *g*. In order to de-lipidise the sample diisopropyl ether was added. After extensive mixing, the sample was spun at 20,000 *g*. The supernatant was covered by a myelin layer. A needle was put through the myelin layer and only the supernatant drawn up into a 1 mL syringe. The myelin layer and pellet were decanted.

### Protein biomarker assays

Levels of NfH phosphoforms, ferritin, GFAP and S100B were quantified using in-house ELISAs techniques as described (Green et al., 1997; Keir et al., 1993; Petzold et al., 2003, 2004). All samples were analysed in duplicates and repeated if the error between duplicates exceeded 10%. Here we adhere to a previously proposed nomenclature where NfH captured by the monoclonal antibody SMI34 is indicated as NfH^*SMI*34^ and captured by the monoclonal antibody SMI35 as NfH^*SMI*35^ (Petzold, 2005; Petzold et al., 2003). The precise binding epitopes of these antibodies are not known. The binding of SMI34 is highly phosphate dependent and therefore related to the degree of NfH phosphorylation. Total protein was determined using the Bio-Rad Protein assay (Bio-Rad, Hemel Hempstead, UK).

### Data analysis

All statistical and data analyses were carried out using SAS (version 9.1, SAS Institute, Inc., Cary, NC, USA). Correlation analysis was performed using the Spearman correlation coefficients followed by the Bonferroni correction in case of multiple analyses. A p-value of < 0.05 was accepted as significant.

## Results

### Patient characteristics and quality of post-mortem material

The mean age of the patients was 56 years (SD: 14 years; range: 34–82); their mean disease duration was 23 years (SD: 10; range: 6–37). Brains were retrieved by the MSTB a mean of 17 h (SD: 6 h; range: 9–28 h) after death. The brain slices were scanned 52 h (SD: 23; range: 23–103) after death.

The individual patients' characteristics are summarised in Table 1. A total of 128 samples were analysed. The number of samples analysed per patient varied from 4 to 18 (Table 1).

Importantly, there were no significant correlations of any of the brain proteins with the delay from death to post-mortem or tissue analysis (Table 2). There were no correlations between brain proteins and the pH of the post mortem CSF. Similarly, brain proteins were not correlated to age, years of disease duration and degree of disability (Table 2). Table 3 summarises the concentration of all protein biomarkers per mg of total soluble protein. The highest concentration was observed for NfH ^*SMI*34^ (median 2.7 μ_?_g/mg protein, calculating to 0.0027% of total soluble protein. The corresponding NLWM T1 relaxation time was 661 ms with a MTR of 34.5 ms.

### Light microscopy

**Fig. 1:**
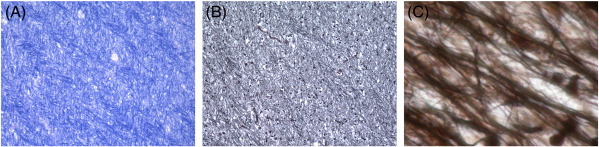
Histology of non-lesional white matter. (A) The Luxol fast blue staining does not reveal any areas of de- or re-myelination of the analysed tissue blocks (×125). (B) There is evidence for irregularities in axonal diameter (Bielschowsky, ×125), (C) better seen at higher magnification (Bielschowsky, ×1250).

Areas from which samples for protein extraction had been obtained were free of de- or remyelinated lesions (Fig. 1A). In this NLWM, some degree of axonal swelling was observed (Fig. 1B). The irregular shape of axons from NLWM becomes more apparent at a higher resolution (shown magnified in Fig. 1C). There was a significant amount of gliosis throughout the analysed NLWM tissue.

### Protein–protein relationship

Strong correlation between NLWM S100B and ferritin levels (R = 0.87, p = 0.0002, data not shown). A weaker correlation was found for NLWM S100B and GFAP levels (R = 0.60, p = 0.03, data not shown). There was no correlation between NfH ^*SMI*34^ and NfH ^*SMI*35^ with each other or any of the other proteins.

### Brain protein–MRI relationship

**Fig. 2:**
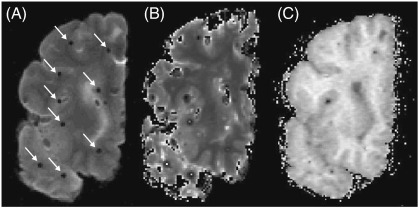
Magnetic resonance imaging of an exemplary post mortem MS brain slice. (A) T_2_ weighted image, (B) T_1_ relaxation map, (C) magnetisation transfer ratio (MTR) map. Arrows indicate artefacts due to tooth picks with which tissue was fixed to a cork board.

**Fig. 3:**
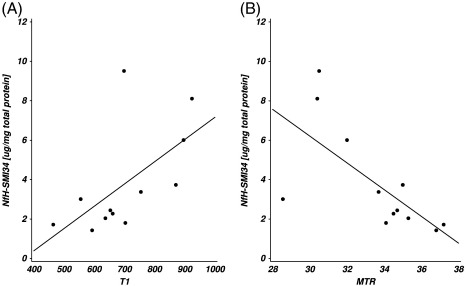
In NLWM (A) NfH^*SMI*34^ was correlated with T_1_ relaxation time (R = 0.70, p = 0.0114) and (B) inversely with magnetisation transfer ratio (MTR, R = − 0.76, p = 0.0065).

Representative 1.5 T MR images of post mortem MS brain slice are shown in Figs. 2A–C. Fig. 2A shows the appearance of artefacts due to fixation of the brain slice on a cork board. These areas were not used for analyses. The quantitative data for the T1 relaxation time and MTR of the NLWM was summarised in Table 3. Significant correlations were detected between NfH ^*SMI*34^ and T1 (R = 0.70, p = 0.01, Fig. 3A) and — inversely — with the MTR (R = − 0.73, p<0.01, Fig. 3B). No correlations were detected between MRI indices and any other of the measured brain proteins.

## Discussion

The main finding of this study was the strong correlation between post-translational modifications of axonal proteins and two quantitative MRI indices in NLWM as both T1 and — inversely — MTR were strongly associated with the tissue concentration of hyperphosphorylated NfH (NfH^*SMI*34^).

The MRI data is consistent with previous studies. In these studies MTR was shown to be a predictor of myelin content in MS brain provided lesions were included in the analysis (Barkhof et al., 2003; Schmierer et al., 2004, 2007, 2008). Earlier evidence that in MS lesions MTR primarily reflects the presence or absence of axons (van Waesberghe et al., 1999; van Walderveen et al., 1998) had been challenged by multi-variate statistical analyses showing that the correlation between MTR and axons was secondary to the primary association between MTR and myelin content (Barkhof et al., 2003; Schmierer et al., 2004). The significant axonal damage and loss in demyelinated MS lesions (Trapp et al., 1998) and hence the marked lesional MTR reduction may however mask detection of more subtle changes in NLWM. Of note, myelin induces and controls Nf phosphorylation (Dewaegh et al., 1992; Petzold, 2005; Sanchez et al., 2000). Therefore the data on the relationship between myelin and MTR (Barkhof et al., 2003; Schmierer et al., 2004) may also be interpreted as indirect evidence of changes in Nf phosphorylation.

Given that MTR changes were also detected in areas that did not show overt demyelination, the suggestion is plausible that a different tissue component (or several thereof) may underlie MTR changes in NLWM. Our data indicate that MTR (and T1) may reflect early post-translational changes of the axonal architecture. An early feature of these changes in MS is phosphorylation of Nf and tau proteins, shown in cell-culture (Jackson et al., 2004), animal models (Anderson et al., 2008; Schneider et al., 2004) and post-mortem tissue (Dutta et al., 2006; Petzold et al., 2008; Trapp et al., 1998).

Irrespective of its specificity for certain tissue features, MTR has been shown to be highly sensitive for subtle tissue alterations in the CNS of pwMS outside MS lesions (Symms et al., 2004). Several groups have reported a mild downward slope of MTR preceding by months the acute blood brain barrier disruption that characterises new demyelinating lesions in patients with MS (Fazekas et al., 2002; Filippi et al., 1998; Goodkin et al., 1998). It is intriguing to hypothesise such early MTR changes result, at least in part, from the switch in the phosphorylation status of axonal neurofilaments as our data suggest.

The correlation between NfH^*SMI*34^ and T1 is consistent with (1) the detected change in MTR in this study and (2) previous work showing loss of compactness and organisation of the axonal cytoskeleton in MS NLWM (Petzold et al., 2008), resulting in a larger free proton pool (thus increasing T1).

**Fig. 4:**
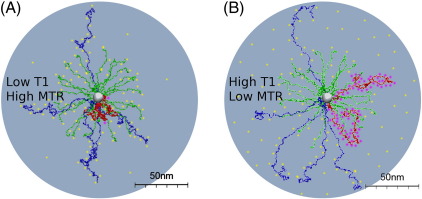
Typically there is a balance between free protons and macromolecular bound protons. It is proposed that this balance is dependent on NfH phosphorylation (pink dots). (A) Negatively charged aminoacids in dephosphorylated NfH (red), NfM (blue) and NfL (green) provide reversible binding sites for a large pool of free protons (yellow dots) which upon magnetic stimulation move from the macromolecular bound to become free protons. This is reflected in a low T_1_ and high MTR (B) For hyperphosphorylated NfH reversible proton binding sites are extensively covered by phosphate (P^4 +^, pink dots). Subsequently there are relatively less protons bound to the semi-solid pool with a larger proportion of free protons. Therefore the T_1_ increases and MTR decreases. The protein-structure model is based on in vivo data from patients with multiple sclerosis and was adapted from (Kim et al., 2011).

Our data on NfH is in line with what others found for tau protein (Anderson et al., 2008; Schneider et al., 2004). Neurofilaments are unique polyampholyte allowing for considerable change of charge and structure (Petzold, 2005). As heavily phosphorylated Nf proteins branch out into the intracellular space of axons this may influence the balance between free and macromolecular bound protons, possibly by charge-repulsion. Taken together the present data suggests that the MRI quantifiable increase of proton mobility in NLWM may be possible intracellularly and due to post-translational modifications of NfH, likely also tau and possibly other proteins. Protein-structurally the polyampholyte NfH should be of particular interest to proton dependent measurements such as MTR or T1. The charge of non-phosphorylated NfH is near neutral (− 2e) with negatively charged amino acids loosely binding protons (Fig. 4A top). In contrast, the charge changes to −82e if fully phosphorylated (NfH^*SMI*34^). Strongly positive charged phosphor (4+) now is bound to the aminoacids in NfH^*SMI*34^. This leads to extension of the NfH sidearms from about 35 nm (Fig. 4A, red chain) to 70 nm from the core at an ionic strength of 10 mM (Fig. 4B, red chain) (Kim et al., 2011). Additionally, the strong charge repulsive interactions between neighbouring sidearm coronas (Beck et al., 2010) may increase the relative proportion of free protons compared to the non–phosphorylated stage (Figs. 4A and B). Biochemically the densely wrapped composition of myelin proteins do not permit such structural changes, nor do myelin proteins have the polyampholyte features of Nf proteins. Additionally, in this study we only investigated light microscopic normal myelinated areas which virtually excludes a relevant drop out of myelin. Instead our data suggests that the phosphorylation related increased proton-binding capacity of axonal proteins such as NfH are likely responsible for the internally consistent changes of MTR and T1.

## Figures and Tables

**Table 1:** Subject characteristics. Duration is the disease duration in years. Interval 1 is time from death to post-mortem and interval 2 time from death to MRI analysis in hours. The number of tissue samples taken per patient is also provided.

Case	Age	Gender	EDSS	Duration	Course	Interval 1 (h)	Interval 2 (h)	Samples
#1	72	F	7	35	5	17.5	41.5	9
#2	49	F	9	14	2	16.5	44.5	18
#3	76	F	8.5	23	6	11	38	11
#4	52	M	7	24	6	13.5	23	11
#5	58	F	9	32	2	16.5	40	9
#6	82	F	8.5	37	2	15	77	8
#7	50	F	7.5	31	2	9	103	9
#8	34	F	9.5	12	2	12	33	4
#9	55	F	6.5	20	2	24	48	7
#10	44	F	9	16	2	18	83	18
#11	45	F	8.5	6	2	28	44	6
#12	56	M	8.5	29	6	25	48.5	18

**Table 2:** Correlation table of brain proteins with demographic data, brain weight, post-mortem CSF pH and time delay from death to analysis (hours). The R-values are shown. The Bonferroni corrected p-value for multiple comparisons (4 comparisons per variable) is 0.0125.

Variable	NfH^*SMI*34^	NfH^*SMI*35^	Ferritin	S100B	GFAP
Age	− 0.21	− 0.03	− 0.69⁎⁎	− 0.48	− 0.30
EDSS	0.19	0.14	0.14	0.04	0.13
Disease duration	− 0.40	− 0.07	− 0.54	− 0.29	0.09
CSF pH	− 0.26	0.25	0.33	0.10	0.30
Brain weight	0.05	0.33	0.35	0.42	0.65⁎
interval 1	0.27	− 0.50	− 0.04	0.00	− 0.49
interval 2	− 0.08	− 0.38	0.15	0.41	0.16

⁎ p *<* 0.05.

⁎⁎ p *<* 0.0125.

**Table 3:** Composition of NLWM.

Variable	Median	IQR
*Brain proteins in μg/mg protein*		
NfH^*SMI*34^	2.7	1.9–4.9
NfH^*SMI*35^	1.6	1.4–1.9
Ferritin	1.6	0.8–2.9
S100B	2.0	1.3–2.9
GFAP	0.8	0.6–1.1

*MRI indices in ms*		
T1 relaxation time	661	593–755
MTR	34.5	32.0–35.3
